# An action decoding framework combined with deep neural network for predicting the semantics of human actions in videos from evoked brain activities

**DOI:** 10.3389/fninf.2025.1526259

**Published:** 2025-02-19

**Authors:** Yuanyuan Zhang, Manli Tian, Baolin Liu

**Affiliations:** School of Computer and Communication Engineering, University of Science and Technology Beijing, Beijing, China

**Keywords:** functional magnetic resonance imaging, decoding, action semantic, three-dimension convolutional neural network, multi-subject model

## Abstract

**Introduction:**

Recently, numerous studies have focused on the semantic decoding of perceived images based on functional magnetic resonance imaging (fMRI) activities. However, it remains unclear whether it is possible to establish relationships between brain activities and semantic features of human actions in video stimuli. Here we construct a framework for decoding action semantics by establishing relationships between brain activities and semantic features of human actions.

**Methods:**

To effectively use a small amount of available brain activity data, our proposed method employs a pre-trained image action recognition network model based on an expanding three-dimensional (X3D) deep neural network framework (DNN). To apply brain activities to the image action recognition network, we train regression models that learn the relationship between brain activities and deep-layer image features. To improve decoding accuracy, we join by adding the nonlocal-attention mechanism module to the X3D model to capture long-range temporal and spatial dependence, proposing a multilayer perceptron (MLP) module of multi-task loss constraint to build a more accurate regression mapping approach and performing data enhancement through linear interpolation to expand the amount of data to reduce the impact of a small sample.

**Results and discussion:**

Our findings indicate that the features in the X3D-DNN are biologically relevant, and capture information useful for perception. The proposed method enriches the semantic decoding model. We have also conducted several experiments with data from different subsets of brain regions known to process visual stimuli. The results suggest that semantic information for human actions is widespread across the entire visual cortex.

## 1 Introduction

In recent years, the brain’s capacity for semantic decoding of visual stimuli has become a popular area in cognitive neuroscience research. The study of semantic decoding of brain activity will not only provide a better understanding of the cognitive mechanism of the brain but also the development of artificial intelligence. Among the many techniques that can be used to measure brain activity, functional magnetic resonance imaging (fMRI) is advantageous given its high spatiotemporal resolution (Engel et al., 1997).

Numerous studies have developed various methods to estimate the semantic information associated with brain activity based fMRI data (Akamatsu et al., 2018; Li et al., 2021; Stansbury et al., 2013; Takada et al., 2020; Vodrahalli et al., 2018). In early studies, statistical methods such as support vector machine (SVM) classifiers (Cortes and Vapnik, 1995) and linear discriminant analysis (LDA) (Fisher, 2012) are used to decode semantic information (Huth et al., 2012; Huth et al., 2016; Stansbury et al., 2013) for direct classification of categories corresponding to fMRI activities. Recent studies incorporating technological advancements in cognitive neuroscience methods have revealed that deep neural network models (DNNs) can partially explain the brain’s responses to visual stimuli (Cichy et al., 2016; Eickenberg et al., 2016; Güçlü and van Gerven, 2015; Yamins et al., 2014). DNNs representations can provide accurate predictions of neural responses in both the dorsal (object recognition) and the ventral (motion processing/recognition) visual pathways. Many decoding studies have utilized DNN representations to construct models for decoding semantic information of both observed (Akamatsu et al., 2020; Horikawa and Kamitani, 2017a; Matsuo et al., 2018; Wen et al., 2018) and imagined (Horikawa and Kamitani, 2017b) picture stimuli based on the brain activities. They establish a regression mapping from fMRI to DNN representations, and convert the predicted representations into semantic tags through the pre-trained classifier. As the classifier is separated from the regression mapping, the model can be expanded by retraining the classifier with labeled images without changing the semantic representation space. Compared with models that directly classify fMRI data, the model based on DNN representations provides an effective extension of decoding capabilities (Wen et al., 2018).

However, these semantic decoding studies have mainly explored the scene/object semantic decoding of perceived images based on DNN representations from fMRI activities (Akamatsu et al., 2020; Horikawa and Kamitani, 2017a; Matsuo et al., 2018). By contrast, only a few studies have investigated the semantic decoding of human actions in videos based on fMRI data, and it is unclear whether and to what extent the DNN could decode the brain’s responses to human actions in video stimuli. Among recent studies, Güçlü and van Gerven (2017) demonstrates that the spatiotemporal features of natural movies extracted by a 3D Convolutional (C3D) network (Tran et al., 2015) optimized for action recognition can accurately predict how the dorsal flow area responds to dynamic changes in natural video stimuli (Güçlü and van Gerven, 2017). This method extends beyond the capabilities of the previous models, which can only learn spatiotemporal representations (Nishimoto and Gallant, 2011; Rust et al., 2005). However, the DNN used in (Güçlü and van Gerven, 2017) still lacks the ability to model long-term dependence. An expanding three-dimensional (X3D) (Feichtenhofer, 2020) deep neural network model has been proposed to expand not only in the temporal dimension, but also in other dimensions such as spatial resolution, frame rate, etc., while being extremely light in terms of network width and parameters. Recently, a vision transformer and a video vision transformer were proposed for image and video recognition with a self-attention mechanism was used to capture the relationship of features globally (Arnab et al., 2021; Dosovitskiy et al., 2021). Although this improves the problem of neglecting global integration, the problem of time-consuming computation becomes more serious since transformers contain many more trainable parameters than CNNs with the same number of layers. In some action recognition tasks, convolutional neural networks such as X3D outperformed the Transformer models (Lai et al., 2023).

Inspired by recent studies, we construct a baseline framework for decoding human action semantics in videos by establishing relationships between brain activities and semantic features of human actions extracted by DNN. We try to determine whether long-term dependent action features and the use of multi-layer features of DNNs could help to improve the mapping from fMRI data to action features. The framework consists of two parts. The first part aims to capture action features containing spatiotemporal dynamic information through an X3D (Feichtenhofer, 2020) deep neural network model. In this part, an non-local attention mechanism (Wang et al., 2018) is added to the X3D deep learning model to extract long-term dependent action features, which helps to overcome the shortcomings of the deep learning model that can not capture features of long-term dependence. The second part aims to build a regression model from fMRI to action features and convert the predicted representations into semantic tags through the pre-trained classifier to decode the action semantics in the videos. In this part, we use multi-layer feature loss constraints as a loss constraint term of the MLP to establish an accurate mapping relationship from fMRI to action features. To demonstrate the advantage of the multi-layer feature loss-constrained MLP approach, we compare its decoding performance with ridge regression, K-nearest neighbor (KNN), and MLP regression approaches, which are various visual techniques widely used in decoding research (Matsuo et al., 2018; Papadimitriou et al., 2019; Qiao et al., 2018; Wen et al., 2018). In this study, we use the fMRI dataset published by Tarhan and Konkle (2020), which consists of fMRI recordings of 13 people watching daily human behavior videos. We also try to perform fMRI data augmentation through linear interpolation to expand the amount of data to improve the decoding effect. Finally, we have conducted several experiments using reliable voxels acquired from the whole cortex and reliable voxels from specific brain regions, to gain further insight into the brain’s understanding of motion video.

## 2 Materials and methods

### 2.1 Overview

**FIGURE 1:**
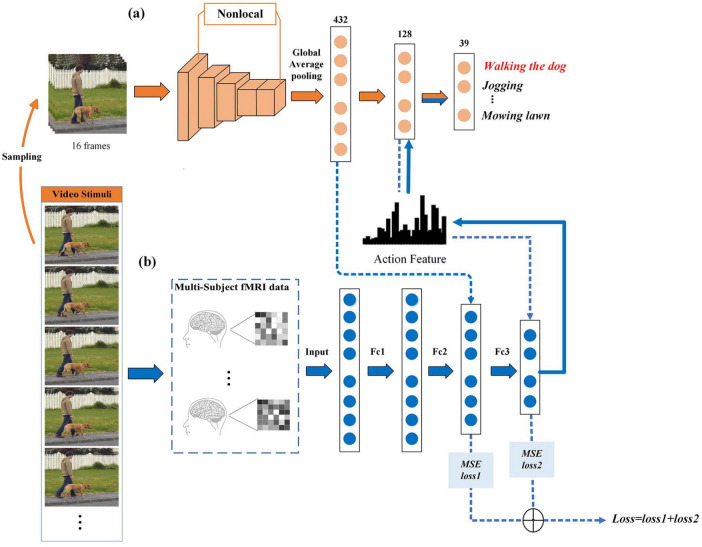
Overview of the proposed method. **(a)** The X3D model. The orange arrows represent the learning process of the deep learning model X3D from video input to output action semantics. **(b)** The MLP regression model. The blue arrows represent the process of predicting action features from fMRI, and then from the predicted features to action semantics. Images were taken from Tarhan and Konkle (2020) (Creative Commons License CC BY 4.0, https://creativecommons.org/licenses/by/4.0/).

Figure 1 summarizes the proposed framework for decoding human action semantics in videos based on fMRI data. The decoding model is divided into two parts: (1) Extracting action features by utilizing the action recognition model, X3D. The X3D model takes a raw video clip as input that is uniformly sampled 16 frames in the data layer stage. The non-local-attention mechanism is added to X3D to capture long-range temporal and spatial dependence so as to overcome the shortcomings of the deep learning model that cannot capture features of long-term dependence. The first fully connected layer of X3D is the action feature extraction layer, and the output of this layer is the action feature corresponding to the video stimulus. (2) The MLP regression model is established with a three-layer fully connected network, mapping from fMRI data to action features. The predicted action features are then fed into the second fully connected layer of the X3D model, which serves as the semantic classification layer as defined by the X3D action recognition model, to extract semantic content. To achieve a more accurate mapping between fMRI and deep learning representations, multiple-layer feature mean square error (MSE) is incorporated into the MLP model’s loss function. The model is trained using multi-subject fMRI data and subsequently tested on an unseen subject to evaluate its generalization capabilities.

### 2.2 Dataset and preprocessing

Functional magnetic resonance imaging data set published by Tarhan and Konkle (2020) is used in the study, which contains information from 13 subjects who have watched videos of typical daily human behavior. The stimuli consist of 120 videos (duration: 2.5 s) reflecting 60 types of daily human movement (e.g., running, cooking, riding a bike), which are obtained from YouTube, Vine, the Human Movement Database (Kuehne et al., 2011) and the University of Central Florida’s Action Recognition Data Set (Soomro et al., 2012). Each video stimulus is 512 × 512 pixels in size and is presented on a 41.5 × 41.5 cm screen, with a viewing angle of approximately 9 × 9 degrees in the participant’s field of view.

The 120 videos are divided into two sets, with each set containing a video for each of the 60 actions. During the experiment, each participant is required to complete eight runs. In each run, participants watch all 60 action videos from one of the two sets. The videos are displayed in a random order, and each 2.5 s video is shown twice consecutively in each run. To avoid visually jarring transitions between video presentations, a 500 ms time window is used for fading in and out to a uniform gray background at the start and end of each presentation, respectively. The fixation period is 4 s at the beginning of the run and 10 s at the end. Additionally, four 15 s blocks of fixation are interspersed throughout the run.

Imaging data are collected using a 3T Siemens Prima functional magnetic resonance imaging scanner. High-resolution T1-weighted anatomical scans are obtained using the 3D MPRAGE protocol [Time of Repetition (TR) = 2,530 ms; Time of Echo (TE) = 1.69 ms; FoV = 256 mm; 1 × 1 × 1 mm voxel resolution; 176 sagittal slices; gap thickness = 0 mm; Flip Angle (FA) = 7°]. Blood oxygenation level-dependent (BOLD) contrast functional scans are obtained using a gradient echo-planar T2* sequence (84 oblique axial slices acquired at a 25° angle off of the anterior commissure-posterior commissure line; TR = 2,000 ms; TE = 30 ms; FoV = 204 mm; 1.5 × 1.5 × 1.5 mm voxel resolution; gap thickness = 0 mm; FA = 80 degrees; multi-band acceleration factor = 3). The collected fMRI data undergo the corresponding preprocessing steps using Brain Voyager QX software, including slice-scan time correction, three-dimensional motion correction, linear trend removal, temporal high-pass filtering (cut-off of 0.008 Hz), spatial smoothing [4 mm full-width at half-maximum (FWHM) kernel], and normalization to Talairach space.

The whole brain random effect general linear models (GLMs) of each participant are applied to each video set, and to the odd and even runs of each video set. In all cases, square-wave regressors for each 5 s stimulus presentation time are convolved with a 2-gamma function approximating the idealized hemodynamic response, and the regressors for each conditional block are included in the design matrix. In these GLMs, the mean variance inflation factor under the design matrix condition is 1.03 (where values greater than five are considered problematic) and the mean efficiency is 0.21. The time series of voxels are z-normalized in each run and corrected for temporal autocorrelation during GLM fitting. And a second-order autoregressive, AR(2), is used in the GLM. Because the reliable coverage of the participants differs, cross-subject comparison is challenging. Therefore, the decoding model is analyzed in the same voxel as that obtained in the random effects group GLM. The experiment selected 39 fMRI data corresponding to action stimulation videos for subsequent analysis.

### 2.3 Reliability voxel selection

**FIGURE 2:**
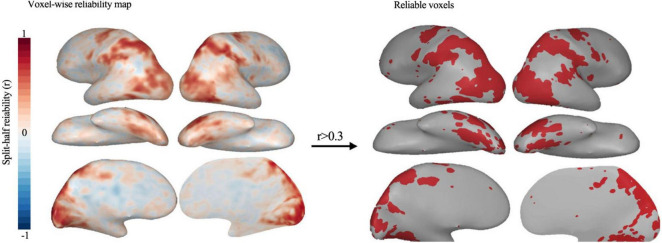
Whole-brain map of split-half voxel reliability. And Reliable voxels (*r* > 0.30) selected based on the Tarhan and Konkle (2019). These results are based on group data.

We adopt the reliable voxels selection method to process the fMRI data and select reliable voxels (Tarhan and Konkle, 2020). The reliability-based voxel selection retains voxels that show systematic differences in activation across the different actions, removing less reliable voxels and voxels that respond equally to all actions. Additionally, this method requires voxels to show similar activation levels across the different actions. Thus, selected voxels necessarily have some tolerance to very low-level features. Split-half reliability is calculated for every voxel by correlating betas extracted from even and odd runs. The reliability is obtained in two ways: The within-set reliability is calculated by correlating the odd and even betas of each set separately, and then the resulting r-maps are averaged. Cross-sets reliability is also calculated by correlating odd and even betas of glms computed on the two video sets. Cross-sets reliable voxels have relatively low reliability in early visual areas, and within-set reliable voxels have better coverage of early visual cortex. For both types of reliability, a procedure from Tarhan and Konkle (2019) is used to select reliability-based cutoffs. First, the reliability of each video’s multi-voxel response pattern is plotted across a range of candidate cutoffs. Then, the cutoff is chosen based on where the multi-voxel mode reliability for all videos starts to stabilize. Using this approach, any voxel with an average reliability of 0.3 or higher is a reasonable cutoff to be included in the feature modeling analysis, as it maximizes reliability without sacrificing too much coverage. This cutoff holds for both group and single-subject data. Finally, these reliable voxels activated (Figure 2) along a broad extension of the ventral and parietal cortices, cover the lateral occipitotemporal cortex (OTC), the ventral OTC and the intra-parietal sulcus (IPS).

In order to clarify the cognitive mechanism of movement understanding in the brain, we select several regions of interest for classification and decoding. According to previous cognitive neuroscience studies on motion perception, human brain motion perception not only involves visual regions, but also involves an Action Observation Network, which is composed of three core regions of occipito-temporal, parietal, and premotor regions in the human brain. The primary motor cortex (Brodmann 4), auxiliary motor cortex/premotor cortex (Brodmann 6), primary visual cortex (Brodmann 17), secondary visual cortex (Brodmann 18) and higher visual cortex (Brodmann 19) are selected as areas of interest for analysis according to the Brodmann template.

### 2.4 Extraction of action features

In this study, the three-dimensional convolutional neural network X3D (Feichtenhofer, 2020) is used to extract action features from human action videos. The model is pre-trained on the large-scale action recognition database Kinetics-400 (Kay et al., 2017). X3D consists of nine layers, the first of which is a three-dimensional convolutional layer. Then, there are four resnet layers, each of which contains 3, 5, 11, and 7 resnet blocks. Each resnet block includes three convolutional layers: 1 × 1 × 1, 3 × 3 × 3, and 1 × 1 × 1 convolution kernel operations. The last four layers are a convolutional layer, a global average pooling layer with an output of 432 dimensions, and two fully connected layers. The output dimensions of the two fully connected layers are 2,048 and 400, respectively, where 2,048 represents the dimension for extracting action features, and 400 represents the number of action categories. The model retains the temporal input resolution of all elements in the entire network hierarchy and does not start temporal downsampling until the global average pooling layer.

Normally, the three-dimension CNN model involves the stacking of spatiotemporal convolution operations. However, Convolution operations can only be local operations in space or time. CNN only captures information in a small neighborhood in time or space, and it is difficult to capture dependence at further locations. To compensate for this limitation of X3D, we incorporate a nonlocal-attention mechanism (Affolter et al., 2020) before the global average pooling layer. The nonlocal-attention mechanism directly captures remote dependencies by calculating the relationship between any two locations, regardless of their distance from one another. In this study, we compare the decoding effect when we add and do not add a nonlocal-attention mechanism in X3D to explore whether long-term spatiotemporal dependencies are more beneficial for decoding.

In addition, due to the small amount of fMRI data, the 2,048-dimensional action features extracted by X3D are not conducive to the establishment of regression mapping. Therefore, we make structure modification of X3D model. We set the output dimensions of the first fully connected layer to 512, 256, and 128, respectively. We then set the output of the last fully connected layer to 39, as 39 semantic categories overlap between our set and the large-scale action recognition database Kinetics. Then we fine-tune the adjusted X3D model. Finally, the output dimension of the penultimate layer is set to 128 through a comparison based on decoding results. To retrain the modified model, we uniformly sample the corresponding 39 semantic categories of partial videos from the Kinetics-400 database. The size of the training dataset is approximately 14,323, while that of the verification dataset is 1,916. Finally, through transfer learning based on the trained X3D model, the action features of the stimuli are extracted directly. The main model structure and model parameters are shown in Table 1.

**TABLE 1:** The model structure of expanding three-dimensional (X3D).

Stage	Filters	Output sizes (T × H × W)
Input	Uniformly sample 16 frames	16 ×224 × 224
Conv1	1 × 3 × 3, 24	16 ×112 × 112
Res2	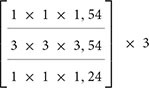	16 ×56 × 56
Res3	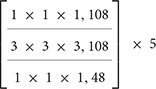	16 ×28 × 28
Res4	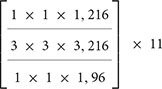	16 × 14 × 14
Res5	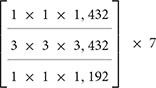	16 × 7 × 7
Conv5	1 ×3 × 3, 432	16 × 7 × 7
Pool5	16 × 7 × 7	1 × 1 × 1
Fc1	1 × 1 × 1, 2,048	1 × 1 × 1
Fc2	1 × 1 × 1,39	1 ×1 × 1

In the model fine-tuning process, 16 frames are uniformly selected from each video for input, and the length and width of each frame are 224 × 224. In the last fully connected layer, the input is the action feature representation, *y*, from the penultimate layer of X3D, and the output is the normalized probability, *q*, by which the action video is classified into each category. The model is trained using the stochastic gradient descent (SGD) to minimize the cross-entropy loss from the predicted probability *q* to the true value *p*. Cross-entropy loss mainly describes the distance between the actual output (probability) and expected output (probability); that is, the smaller the value of the cross-entropy loss, the closer the two probability distributions are. The predicted probability is expressed as:


(1) $$\begin{array}{c} q=\frac{e^{W\,y+b}}{\sum_{j=0}^{N}e^{W\,y+b}} \end{array}$$

where *N* represents the number of all categories, *W* and *b* represent the weight and bias. To fine-tune the X3D model, the minimized cross-entropy loss *H* is expressed as follows:


(2) $$H\left(p,\;q\right)\;=\;-\sum_{x}p\left(x\right)logq\left(x\right)$$

When fine-tuning the model, the batchsize is set to 8, and the learning rate is set to 0.005. After training for 15 epochs, the optimal model on the validation set is selected as the final model, and then is directly migrated to perform feature extraction on the stimuli.

### 2.5 Model for decoding action semantics based on fMRI

The decoding step is mainly divided into two steps: (1) establishing the regression model from the fMRI data to the action features; (2) inputting the predicted action feature into the semantic classification model, which was predefined by the X3D action recognition model, to obtain semantic content. In addition, we directly adopt a data-driven approach to achieve brain decoding for unseen subjects, train the regression model on n-1 subjects, and verify it using data from one subject. The final decoding result is obtained by performing the leave-one-subject-out cross-validation. This setup of decoding for unseen subjects is remarkably challenging, since fMRI data are very different across subjects, among other reasons, owing to a lack of alignment and variable numbers of voxels between subjects (Affolter et al., 2020).

In this study, we first use the principal component analysis (PCA) approach to extract principal components on the obtained 43,949 dimension gray matter data of fMRI. The PCA is used to extract 1,000 principal components from fMRI data, following which a regression mapping model is established between the reduced fMRI data and the action features. Due to the small amount of fMRI data, we hope to introduce X3D’s multi-layer features as an auxiliary task to help the learning process of regression model, so that the model gradually approaches the X3D action semantic space. To achieve this goal, we propose a multi-task loss constraint MLP approach of three-layer fully connected layers with 1,000, 432, and 128 dimensions, respectively. And we additionally add the X3D’s penultimate layer feature constraints as another loss term of regression model. Except for the last layer, we add batch normalization and dropout at a rate of 0.4 at the end of each layer to prevent overfitting. The mean square error (MSE) is then used as the final measurement standard, and the loss function is minimized to update the parameters of the three-layer fully connected network model:


(3) $$L_{loss}\;=\;\frac{1}{n}\;\left(w_{0}\sum_{i=0}^{n}\;{\left(y_{0}-{\hat{y}}_{0}\right)}^{2}\;+\;w_{1}\sum_{i=0}^{n}\;{\left(y_{1}-{\hat{y}}_{1}\right)}^{2}\right)$$

where *n* represents the number of all samples, *y*_0_ and *y*_1_ represent the true value of the output of the last one layer and the penultimate layer, respectively, and ${\hat{y}}_{0}$ and ${\hat{y}}_{1}$ represent the predicted values. *w*_0_ and *w*_1_ represent the weights of the first and second loss items, respectively, and the final values are determined to be 2.2 and 1, respectively according to the accuracy of semantic decoding on the verification set. In the process of training the three-layer fully connected layer, we use the SGD optimizer, the batchsize is set to 16, and the learning rate is set to 0.0001. After 1,000 epochs, training stops.

To evaluate the classification accuracy, we use the prediction accuracy of Top-1 and Top-5. Specifically, for any given action video, we rank the action semantic categories in descending order of probability estimated by fMRI. If the true category is the top 1 of the ranked categories, it is considered to be Top-1 accurate. If the true category is in the top 5 of the ranked categories, it is considered to be Top-5 accurate. In addition, we also use the pairwise classification criterion (Affolter et al., 2020; Papadimitriou et al., 2019) to obtain whether the regression mapping model can establish an accurate mapping relationship from fMRI to action features. For each video, we compute the correlation between the predicted vector and the actual vector. If the predicted vectors are more similar to their corresponding action features than to the alternatives, the decoding is deemed correct. The random baseline is 50%.

### 2.6 Data enhancement

Based on the Mixup method idea (Zhang et al., 2018), we propose a data enhancement approach, which linearly interpolates the different subjects’ data corresponding to the same category to generate new fMRI data and target vector. The Mixup method is a simple method for data enhancement that is independent of the data. It constructs a new sample by linearly interpolating two random samples and their target vectors in the training set. Based on this idea, we first use the data of n-1 subjects to perform PCA so as to extract principal components on the gray matter data of fMRI. And then we linearly weight the different subjects’ principal component features of fMRI data corresponding to the same category to generate new subject data. Both the augmented data and the original data have been tested on test sets.


(4) $$d=\lambda \,d_{s_{i}}+\left(1-\lambda \right)\,d_{s_{j}}$$

(5) $$t=\lambda \,t_{s_{i}}+\left(1-\lambda \right)\,t_{s_{j}}$$

Among them, *d* represents the newly generated fMRI data, *d_s_i__* and *d_s_j__* correspond to two data randomly selected for a certain category. *t* represents the newly generated target vectors, *t_s_i__* and *t_s_j__* correspond to the target vector of *d_s_i__* and *d_s_j__*. λ represents the weight, which is a parameter that obeys the *B* distribution λ*Beta*(α,α). Through training, the final selection value of λ is 0.2.

## 3 Results

### 3.1 Cross-subject decoding

**FIGURE 3:**
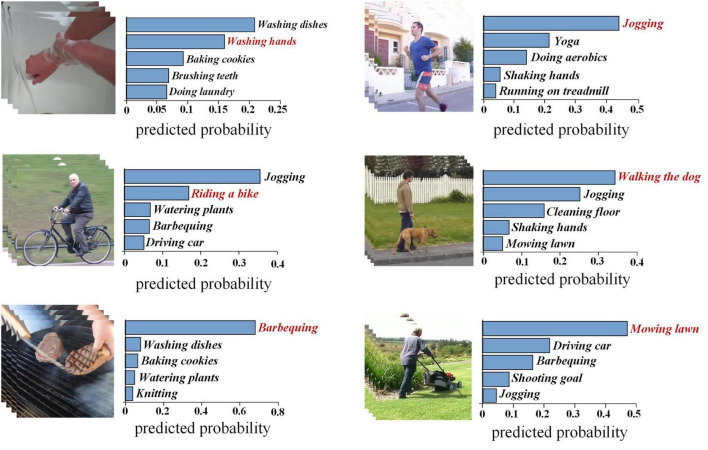
Top-5 estimated semantic categories of human action videos. The vertical line shows the top-5 categories determined from fMRI activity which are shown in the order of descending probabilities from the top to the bottom. The horizontal one shows predicted probability of estimated categories, and correct categories are highlighted in red. Images were taken from Tarhan and Konkle (2020) (Creative Commons License CC BY 4.0, https://creativecommons.org/licenses/by/4.0/).

We first construct a baseline decoding framework that does not add any other modules, that is, we extract action features from videos based on the X3D, and then an MLP model which uses fMRI signals to predict the action features is built, finally the built-in transformation of the predicted features to the last layer (or output layer) of X3D is used to estimate the classification probability. The final assessment of the results is performed by the leave-one-subject-out approach. Figure 3 shows Top-5 predicted categories of some samples. The decoded categories are sorted in descending order of predicted probability. Correct categories are highlighted in red.

**FIGURE 4:**
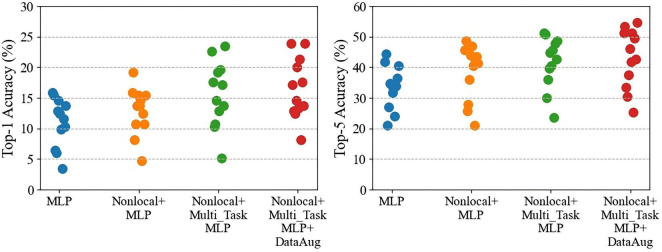
The performance comparisons of Top-1 and Top-5 after gradually adding different modules to the baseline model. Each point represents a subject.

As shown in Table 2, the baseline decoding accuracies of Top-1 and Top-5 are 11.00% (random level 2.56%) and 33.53% (random level 12.82%), respectively, both significantly exceeding the random level. These results show that the human brain has a wealth of representation space for action semantic. At the same time, similar to the literature (Güçlü and van Gerven, 2017), the brain activities are related to the representations extracted by 3D deep learning model for action recognition. Its achieved Top-1 accuracy on average reaches 16.56% and Top-5 accuracy reaches 43.13% by adding three different modules. The reason for this improved result is that the decoding accuracy of each subject has increased overall, and the generalization of the model to the unseen subjects has enhanced by adding three different modules (Figure 4).

**TABLE 2:** Ablation study. Top-1 and Top-5 accuracy after incrementally adding the nonlocal attention mechanism, multi-task multilayer perceptron (MLP) and data augmentation modules to our baseline model.

Model	Top-1 accuracy	Top-5 accuracy
Baseline	11.00%	33.51%
Baseline+Nonlocal	12.86%	38.64%
Baseline+Nonlocal+multi-task MLP	15.56%	41.81%
Baseline+Nonlocal+multi-task MLP+Data Augmentation	16.56%	43.13%

### 3.2 Comparing algorithmic choices

In this section, we mainly compare the contributions of the nonlocal-attention mechanism, multi-task loss constraints regression method, and data enhancement to the decoding model.

#### 3.2.1 Nonlocal-X3D vs. X3D

To compare whether long-range dependent action features are more helpful for decoding. In the experiments, we add a nonlocal attention mechanism to the X3D model and compare the decoding accuracy produced by adding and not adding a nonlocal attention mechanism to the X3D model, thereby determining whether the captured long-distance dependent features are more conducive to decoding. In addition, we also compare the decoding effect by using different deep learning models to extract action features. These models are Inflated 3D Convolutional (I3D) network (Carreira and Zisserman, 2017) and 3D Convolutional (C3D) network (Tran et al., 2015). Güçlü and van Gerven (2017). used the C3D model to extract the DNN representation of the movie, and established the mapping relationship between the backflow region and the DNN representation to identify different movie stimuli. I3D and X3D are the latest deep learning models for action recognition, especially X3D has higher action recognition accuracy. However, C3D and I3D still lack the ability to model long-term dependence.

We use the MLP model to construct regression maps from fMRI to action features and obtain the corresponding results of semantic decoding. As shown in Table 3, these results indicate that the Top-1 accuracy of the X3D model with a nonlocal attention mechanism is 12.86%, and the Top-5 accuracy is 38.64%. Compared with not joining the nonlocal attention mechanism, it has increased by 1.61 and 3.95%, respectively. In addition, the Top-1 decoding accuracies of C3D and I3D models are 8.51, and 6.09%, respectively, and the Top-5 accuracies are 30.66, and 22.69%, respectively. The results show that the decoding accuracy of the action features extracted based on the X3D model is higher.

**TABLE 3:** The impact of different models for extracting action features on the accuracy results of Top-1 and Top-5.

Model for extracting action features	Top-1 accuracy	Top-5 accuracy
Inflated 3D Convolutional network (I3D) (Carreira and Zisserman, 2017)	6.09%	22.69%
3D Convolutional network (C3D) (Tran et al., 2015)	8.51%	30.66%
Expanding three-dimensional network (X3D) (Feichtenhofer, 2020)	11.00%	33.51%
X3D+Nonlocal (ours)	12.86%	38.64%

#### 3.2.2 Comparison of regression mapping models

To place our model in the context of existing work, we compare with three competing approaches: ridge regression, KNN and MLP regression, which are used by previous decoding studies (Matsuo et al., 2018; Wen et al., 2018) in constructing fMRI-to-deep learning features. The three approaches are implemented by using the scikit-learn platform. These three approaches are used for mapping based on action features extracted by X3D with a nonlocal attention mechanism, following which we compare the decoded results. In the experimental settings, the ridge regression regularization parameter alpha is set to 0.2, the NEIGHBORS parameter of KNN is set to 7, and the DEGREE parameter is set to 2. The nonlinear layer of the MLP adopts the relu activation function. And to prevent overfitting during regression mapping learning, the dropout is set to 0.4, the learning rate is set to 0.0001, and the batch size is set to 16. We have tried to build a deeper network, but the overfitting is serious.

**FIGURE 5:**
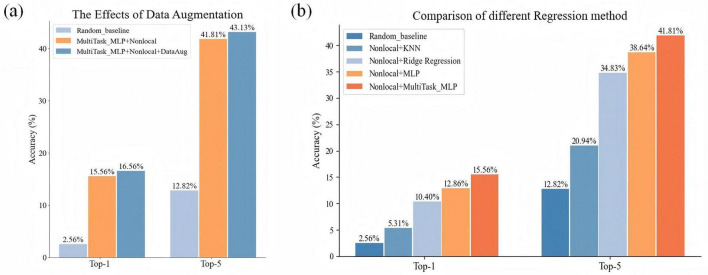
Comparison of the results of adding different modules. **(A)** Comparison of the accuracy results of Top-1 and Top-5 with and without data enhancement. **(B)** The impact of different regression models on the accuracy results of Top-1 and Top-5.

**FIGURE 6:**
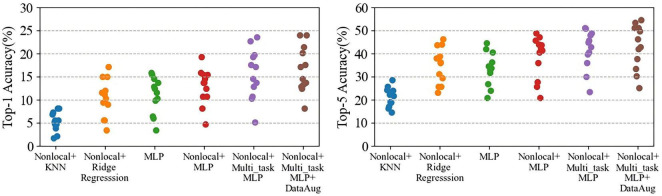
The performance comparisons of Top-1 and Top-5 by using different regression models. Each dot represents a subject decoding performance.

As shown in Figure 5B, the comparison results of multiple models show that our model has 4.30% Top-1 improvement and 5.84% Top-5 improvement compared to ridge regression; 10.58% Top-1 and 20.58% Top-5 improvement compared to KNN; and compared to MLP it has an increase of 2.77% Top-1 accuracy and 3.74% Top-5 accuracy. And by using multi-task MLP regression mapping, the decoding accuracy of each subject is generally improved (Figure 6). The pairwise classification corresponding to KNN, ridge regression, MLP and multi_task MLP are 62.45, 78.74, 81.91, and 83.46%, respectively. Thus showing that Multi_task MLP can more accurately predict action features from fMRI.

#### 3.2.3 Data augmentation effect

Data augmentation is based on the Mixup method and then a linearly weighted combination of data from different subjects corresponding to the same category. This method not only increases the size of the dataset but also improves the generalization of the data. Figure 5A shows that using data augmentation significantly improves decoding results. When compared with the non-enhanced dataset (Top-1: 15.56%, Top-5: 41.81%), the enhanced dataset increased Top-1 and Top-5 accuracy by 1% and 1.32%, respectively.

### 3.3 Cross-subject decoding based reliable voxels

In order to analyze the brain’s cognitive mechanism of action videos, we decode by identifying voxels that can reliably distinguish different actions. We use the X3D model combined with the nonlocal attention mechanism to extract action features, and then predict action features based on reliable voxels to decode action semantics. The results show that the Top-1 and Top-5 accuracy of action semantic decoding based on within-set reliable voxels are 14.89 and 39.78%, respectively. The Top-1 and Top-5 accuracy of action semantic decoding based on cross-sets reliable voxels are 14.10 and 40.42%, respectively. The pairwise classification results based on within-set reliable voxels and cross-sets reliable voxels are 83.03 and 82.34%, respectively. The results show that the Top-1 and Top-5 accuracy of action semantic decoding based on finally whole-brain reliable voxels are 16.27 and 43.52%, respectively.

**FIGURE 7:**
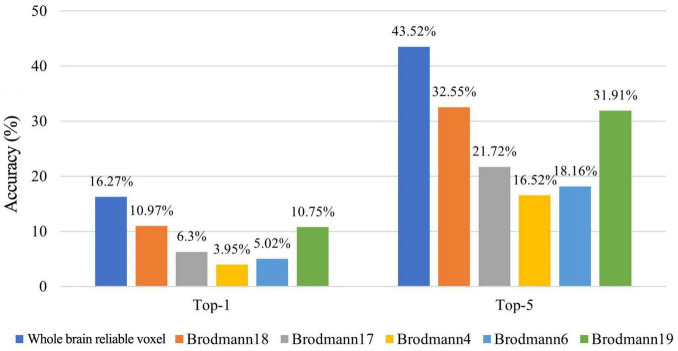
The Top-1 and Top-5 accuracy of action semantic decoding based on different brain regions.

The experimental results in Figure 7 show that the decoding accuracy of each ROI is significantly higher than the random level. In particular, Brodmann 18 and Brodmann 19 (high vision area) have better decoding accuracy. Moreover, the whole-brain reliable voxel-based semantic decoding method achieves higher decoding accuracy than ROI alone, which indicates that the whole-brain reliable voxel-based semantic decoding method in this research does not rely too much on the application of domain-specific knowledge.

## 4 Discussion

Here, we develop a framework for decoding human action semantics based on fMRI data by establishing a corresponding relationship between the action features extracted from video data and fMRI data. The decoding results of Top-1 and Top-5 accuracy both significantly exceed chance levels which shows the feasibility of extracting action-related semantic information in videos.

Compared to the models used in most research on action perception, our model can be used to perform semantic decoding across participants, making it more general and transferable than traditional methods such as multivariate pattern analysis (MVPA). When extracting action features, the model in our research based on X3D-DNN more accurately captures motion-selective receptive fields for action semantic decoding by extracting spatiotemporal video features rather than separate spatial features (Nishimoto et al., 2011; Rust et al., 2005). This is different from previous video decoding frameworks, which previously extracted spatial features of video frames based on CNN (Wen et al., 2018) and focused on object semantic decoding using features extracted by DNN optimized for object recognition (Horikawa and Kamitani, 2017b; Wen et al., 2018). In addition, the Recurrent Neural Network (RNN) (Medsker and Jain, 2001) or Long Short-Term Memory (LSTM) model (Hochreiter and Schmidhuber, 1997) can also process time-series related data and LSTMs are more capable of capturing longer-range dependencies than RNNs, which can be used as an alternative to 3D CNN to extract action features. We further add nonlocal-attention mechanism to the DNN model to proof that the extracted long-range dependent action features are more in line with the cognitive mechanism of the human brain. Furthermore, a recent study proposed the Human-Centric Transformer (HCTransformer), which develops a decoupled human-centric learning paradigm to explicitly concentrate on human-centric action cues in domain-variant video feature learning (Lin et al., 2025). In future research, we will further utilize action features that are more aligned with human cognitive mechanisms to assist in decoding behavioral semantics from brain activity. When attempting to decode action semantics, the most important requirement is the development of an accurate mapping model between fMRI voxels and action features, which allows for more accurate decoding of semantic information related to actions. Compared with KNN, ridge regression (Wen et al., 2018), and traditional MLP methods (Matsuo et al., 2018; Papadimitriou et al., 2019), MLP models based on multi-layer feature constraints can more accurately establish the mapping relationship between fMRI and action features. Multilayer feature constraints can assist the learning process of fMRI to action features, making its distribution closer to that of action features.

In most cases, only a small amount of fMRI data can be acquired in a single subject. Building models from multiple subject’s data and transferring them to test subject data is the key to improving the utility of cognitive neuroscience (Gabrieli et al., 2015). Our results indicate that a multi-subject decoding model based on the whole-brain common representation space can predict unseen individual subjects, which is of great significance. However, the common representation space of different brain regions has different contribution in semantic decoding. If the brain regions can be more accurately located about which brain areas contribute more to the decoding of the multi-subject model and which regions can more accurately extract abstract information related to action semantics according to the decoding accuracy, it will be able to gain a deeper understanding of brain mechanisms.

In order to analyze the brain cognitive mechanism of action videos, we conduct semantic decoding experiments using within-set reliable voxels obtained by correlating the odd and even betas of each set and cross-sets reliable voxels obtained by correlating odd and even betas of glms computed on the two video sets. The decoding results of Top-1, Top-5 and pairwise classification accuracy based on within-set reliable voxels and cross-set reliable voxels are all significantly higher than random levels. The Top-1 and pairwise classification accuracy of within-set reliable voxel decoding is slightly higher than that of cross-set reliable voxel decoding. And the within-set reliable voxels have relatively higher coverage of the early visual cortex than the cross-set reliable voxels. This reveals early visual regions are correlated with action semantic features. It has been shown that the layer depth of the optimal encoding layer of the deep learning model is positively correlated with V1, V2, V3 in the early visual area and the position of the MT in the dorsal flow area (Güçlü and van Gerven, 2017). The decoding results based on different brain regions show that the visual cortex can effectively decode the semantic information of action, and the decoding accuracy of the higher visual cortex is higher than that of the primary visual cortex. Similar to the results of Urgen et al. (2019), the visual region can have a good similarity to the visual computational model. And higher visual areas are associated with higher semantic features than the primary visual cortex.

The lower visual cortex is mainly responsible for receiving and initially processing visual information, such as detecting lines, contours, colors, etc., while the higher visual cortex is responsible for more complex visual processing, such as object recognition and spatial cognition. Accurate recognition of actions is a highly challenging task due to cluttered backgrounds, occlusions, and viewpoint variations, etc. Primary visual features may be less capable of reflecting differences between action categories. Additionally, our approaches differ in the granularity at voxel fitting models. Huth et al. (2012) constructed a semantic space containing action categories which suggested that a voxel’s response could be fit by putting weights on over 1,000 predictors, including verbs like “cooking,” “talking,” and “crawl.” Category labels need to be manually labeled. Our approaches uses features learned from natural images, which indicates that the features in the X3D-DNN are biologically relevant, and capture information useful for perception. The features we used my contain more abstract information and this level of representation may therefore be more appropriate for characterizing the response tuning of mid-to-high-level visual cortex. Future work on analyzing the features extracted from each layer of the model can further explore their correspondence with brain regions.

In addition to improving the deep learning model, data enhancement can be used to improve decoding accuracy. Data augmentation constructs new samples by interpolating the fMRI vectors of two random subjects under the same class and their corresponding target vectors. Data augmentation intuitively extends the distribution of a given training set by providing successive data samples for different target vectors, thus making the network more robust in the test phase. Although we perform data augmentation through linear interpolation and improve decoding accuracy, the improvement does not seem to be significant. Recently, deep learning models such as deep recurrent variational auto-encoder and Generative adversarial networks (GAN) have been used to generate EEG or fMRI data with remarkable results (Panwar et al., 2020; Qiang et al., 2021). But the training of such complex models with small samples is still a challenge. On the basis of simply increasing the sample size using our data augmentation method, future research will explore more complex and effective data augmentation models.

## 5 Conclusion

In this paper, we explore the possibility of action semantic decoding based on fMRI data. We construct a more extensible model based on the action representations extracted by a three-dimensional DNN. The difference between this model and the previous models based on deep learning representation is that it uses a three-dimensional DNN model to extract spatiotemporal dynamic features to establish a connection with fMRI, instead of only extracting spatial image features. The model first extracts action features based on the three-dimensional action recognition model X3D, and an MLP model is built to establish the relationship between fMRI and action features so as to decode the action semantics corresponding to brain activities. Considering that it is difficult to obtain single subject data, the model uses multi-subject data for training and tests on unseen subjects. The final results significantly exceed the random level. In addition, the decoding results are further improved by adding a nonlocal attention mechanism, multi-task loss constraint’s MLP model and data enhancement. Moreover, by examining the results obtained from models trained with data from different regions of the cortex, our results suggest that semantic information for human actions is widespread across the entire visual cortex.

## Data Availability

The original contributions presented in this study are included in the article/supplementary material, further inquiries can be directed to the corresponding author.

## References

[B1] Affolter N. Egressy B. Pascual D. Wattenhofer R. (2020). “Brain2Word: Improving brain decoding methods and evaluation,” in *Proceedings of the Medical Imaging Meets Neurips Workshop-34th Conference on Neural Information Processing Systems*, (NeurIPS).

[B2] Akamatsu Y. Harakawa R. Ogawa T. Haseyama M. (2018). “Estimation of viewed image categories via CCA using human brain activity,” in *Proceedings of the 2018 IEEE 7th Global Conference on Consumer Electronics (GCCE)*, (Piscataway, NJ: IEEE), 202–203.

[B3] Akamatsu Y. Harakawa R. Ogawa T. Haseyama M. (2020). “Multi-view bayesian generative model for multi-subject fmri data on brain decoding of viewed image categories,” in *Proceedings of the IEEE International Conference on Acoustics, Speech and Signal Processing (ICASSP)*, (Piscataway, NJ: IEEE), 1215–1219.

[B4] Arnab A. Dehghani M. Heigold G. Sun C. Lukiæ M. Schmid C. (2021). “Vivit: A video vision transformer,” in *Proceedings of the IEEE/CVF International Conference on Computer Vision*, (Piscataway, NJ: IEEE), 6836–6846.

[B5] Carreira J. Zisserman A. (2017). “Quo vadis, action recognition? A new model and the kinetics dataset,” in *Proceedings of the Inernational Conference on Computer Vision and Pattern Recognition*, (Piscataway, NJ: IEEE).

[B6] Cichy R. M. Khosla A. Pantazis D. Torralba A. Oliva A. (2016). Comparison of deep neural networks to spatio-temporal cortical dynamics of human visual object recognition reveals hierarchical correspondence. *Sci. Rep.* 6 1–13. 10.1038/srep27755 27282108 PMC4901271

[B7] Cortes C. Vapnik V. (1995). Support-vector networks. *Mach. Learn.* 20 273–297.

[B8] Dosovitskiy A. Beyer L. Kolesnikov A. Weissenborn D. Zhai X. Unterthiner T. (2021). An image is worth 16x16 words: Transformers for image recognition at scale. *arXiv [Preprint]* 10.48550/arXiv.2010.11929

[B9] Eickenberg M. Gramfort A. Varoquaux G. Thirion B. (2016). Seeing it all: Convolutional network layers map the function of the human visual system. *NeuroImage* 152 184–194. 10.1016/j.neuroimage.2016.10.001 27777172

[B10] Engel S. A. Glover G. H. Wandell B. A. (1997). Retinotopic organization in human visual cortex and the spatial precision of functional MRI. *Cereb. Cortex* 7 181–192. 10.1093/cercor/7.2.181 9087826

[B11] Feichtenhofer C. (2020). “X3D: Expanding architectures for efficient video recognition,” in *Proceedings of the IEEE Conference on Computer Vision and Pattern Recognition (CVPR)*, (Piscataway, NJ: IEEE), 203–213.

[B12] Fisher R. A. (2012). The use of multiple measurements in taxonomic problems. *Ann. Hum. Genet.* 7 179–188.

[B13] Gabrieli J. D. Ghosh S. S. Whitfield-Gabrieli S. (2015). Prediction as a humanitarian and pragmatic contribution from human cognitive neuroscience. *Neuron* 85 11–26. 10.1016/j.neuron.2014.10.047 25569345 PMC4287988

[B14] Güçlü U. van Gerven M. A. (2015). Deep neural networks reveal a gradient in the complexity of neural representations across the ventral stream. *J. Neurosci.* 35 10005–10014. 10.1523/JNEUROSCI.5023-14.2015 26157000 PMC6605414

[B15] Güçlü U. van Gerven M. A. (2017). Increasingly complex representations of natural movies across the dorsal stream are shared between subjects. *NeuroImage* 145 329–336. 10.1016/j.neuroimage.2015.12.036 26724778

[B16] Hochreiter S. Schmidhuber J. (1997). Long short-term memory. *Neural Comput.* 9 1735–1780. 9377276 10.1162/neco.1997.9.8.1735

[B17] Horikawa T. Kamitani Y. (2017a). Generic decoding of seen and imagined objects using hierarchical visual features. *Nat. Commun.* 8:15037. 10.1038/ncomms15037 28530228 PMC5458127

[B18] Horikawa T. Kamitani Y. (2017b). Hierarchical neural representation of dreamed objects revealed by brain decoding with deep neural network features. *Front. Comput. Neurosci.* 11:4. 10.3389/fncom.2017.00004 28197089 PMC5281549

[B19] Huth A. G. Lee T. Nishimoto S. Bilenko N. Y. Vu A. T. Gallant J. L. (2016). Decoding the semantic content of natural movies from human brain activity. *Front. Syst. Neurosci.* 10:81. 10.3389/fnsys.2016.00081 27781035 PMC5057448

[B20] Huth A. G. Nishimoto S. Vu A. T. Gallant J. L. (2012). A continuous semantic space describes the representation of thousands of object and action categories across the human brain. *Neuron* 76 1210–1224. 10.1016/j.neuron.2012.10.014 23259955 PMC3556488

[B21] Kay W. Carreria J. Simonyan K. Zhang B. Hillier C. Vijayanarasimhan S. (2017). The kinetics human action video dataset. *arXiv [Preprint]* 10.48550/arXiv.1705.06950

[B22] Kuehne H. Jhuang H. Garrote E. Poggio T. Serre T. (2011). “HMDB: A large video database for human motion recognition,” in *Proceedings of the IEEE International Conference Computer Vision*, (Piscataway, NJ: IEEE), 2556–2563. 10.1109/TIP.2019.2952088

[B23] Lai D. K. H. Cheng E. S. So B. P. Mao Y. J. Cheung S. M. Cheung D. S. (2023). Transformer models and convolutional networks with different activation functions for swallow classification using depth video data. *Mathematics* 11:3081.

[B24] Li D. Du C. Wang S. Wang H. He H. (2021). Multi-subject data augmentation for target subject semantic decoding with deep multi-view adversarial learning. *Inf. Sci.* 547 1025–1044.

[B25] Lin K. Y. Zhou J. Zheng W. S. (2025). Human-centric transformer for domain adaptive action recognition. *IEEE Trans. Pattern Anal. Mach. Intell.* 47 679–696. 10.1109/TPAMI.2024.3429387 39012755

[B26] Matsuo E. Kobayashi I. Nishimoto S. Nishida S. Asoh H. (2018). “Describing semantic representations of brain activity evoked by visual stimuli,” in *Proceedings of the 2018 IEEE International Conference on Systems, Man, and Cybernetics (SMC)*, (Piscataway, NJ: IEEE), 576–583.

[B27] Medsker L. R. Jain L. C. (2001). Recurrent neural networks. *Design Appl.* 5 64–67.

[B28] Nishimoto S. Gallant J. L. (2011). A three-dimensional spatiotemporal receptive field model explains responses of area MT neurons to naturalistic movies. *J. Neurosci.* 31 14551–14564. 10.1523/JNEUROSCI.6801-10.2011 21994372 PMC3338855

[B29] Nishimoto S. Vu A. T. Naselaris T. Benjamini Y. Yu B. Gallant J. L. (2011). Reconstructing visual experiences from brain activity evoked by natural movies. *Curr. Biol.* 21 1641–1646. 10.1016/j.cub.2011.08.031 21945275 PMC3326357

[B30] Panwar S. Rad P. Jung T. P. Huang Y. (2020). Modeling EEG data distribution with a wasserstein generative adversarial network to predict rsvp events. *IEEE Trans. Neural Syst. Rehabil. Eng.* 28 1720–1730. 10.1109/TNSRE.2020.3006180 32746311

[B31] Papadimitriou A. Passalis N. Tefas A. (2019). Visual representation decoding from human brain activity using machine learning: A baseline study. *Patt. Recognit. Lett.* 128 38–44.

[B32] Qiang N. Dong Q. Liang H. Ge B. Zhang S. Sun Y. (2021). Modeling and augmenting of fMRI data using deep recurrent variational auto-encoder. *J. Neural Eng*. 18:0460b6. 10.1088/1741-2552/ac1179 34229310

[B33] Qiao K. Zhang C. Wang L. Chen J. Zeng L. Tong L. (2018). Accurate reconstruction of image stimuli from human functional magnetic resonance imaging based on the decoding model with capsule network architecture. *Front. Neuroinformatics* 12:62. 10.3389/fninf.2018.00062 30294269 PMC6158374

[B34] Rust N. C. Schwartz O. Movshon J. A. Simoncelli E. P. (2005). Spatiotemporal elements of macaque v1 receptive fields. *Neuron* 46 945–956. 15953422 10.1016/j.neuron.2005.05.021

[B35] Soomro K. Zamir A. R. Shah M. (2012). UCF101: A dataset of 101 human actions classes from videos in the wild. *arXiv [Preprint]* 10.48550/arXiv.1212.0402

[B36] Stansbury D. E. Naselaris T. Gallant J. L. (2013). Natural scene statistics account for the representation of scene categories in human visual cortex. *Neuron* 79 1025–1034. 10.1016/j.neuron.2013.06.034 23932491 PMC5464350

[B37] Takada S. Togo R. Ogawa T. Haseyama M. (2020). “Generation of viewed image captions from human brain activity via unsupervised text latent space,” in *Proceedings of the 2020 IEEE International Conference on Image Processing (ICIP)*, (Piscataway, NJ: IEEE), 2521–2525.

[B38] Tarhan L. Konkle T. (2019). Reliability-based voxel selection. *NeuroImage* 207:116350. 10.1016/j.neuroimage.2019.116350 31733373

[B39] Tarhan L. Konkle T. (2020). Sociality and interaction envelope organize visual action representations. *Nat. Commun.* 11:3002. 10.1038/s41467-020-16846-w 32532982 PMC7293348

[B40] Tran D. Bourdev L. Fergus R. Torresani L. Paluri M. (2015). “Learning spatiotemporal features with 3D convolutional networks,” in *Proceedings of the IEEE International Conference on Computer Vision*, (Piscataway, NJ: IEEE).

[B41] Urgen B. A. Pehlivan S. Saygin A. P. (2019). Distinct representations in occipito-temporal, parietal, and premotor cortex during action perception revealed by fMRI and computational modeling. *Neuropsychologia* 127 35–47. 10.1016/j.neuropsychologia.2019.02.006 30772426

[B42] Vodrahalli K. Ko J. Chiou A. Novoa R. Abid A. Phung M. (2018). Mapping between fMRI responses to movies and their natural language annotations. *NeuroImage* 180 223–231. 10.1016/j.neuroimage.2017.06.042 28648889 PMC5742073

[B43] Wang X. Girshick R. Gupta A. He K. (2018). “Non-local neural networks,” in *Proceedings of the IEEE Conference on Computer Vision and Pattern Recognition (CVPR)*, (Piscataway, NJ: IEEE), 7794–7803. 10.3390/bioengineering11060627

[B44] Wen H. Shi J. Zhang Y. Lu K. H. Cao J. Liu Z. (2018). Neural encoding and decoding with deep learning for dynamic natural vision. *Cereb. Cortex* 28 4136–4160. 29059288 10.1093/cercor/bhx268 PMC6215471

[B45] Yamins D. L. Hong H. Cadieu C. F. Solomon E. A. Seibert D. DiCarlo J. J. (2014). Performance-optimized hierarchical models predict neural responses in higher visual cortex. *Proc. Natl. Acad. Sci.* 111 8619–8624. 10.1073/pnas.1403112111 24812127 PMC4060707

[B46] Zhang H. Cisse M. Dauphin Y. N. Lopez-Paz D. (2018). mixup: Beyond empirical risk minimization. *arXiv [Preprint]* 10.48550/arXiv.1710.09412

